# Draft Genome Sequences of *Geomicrobium* sp. Strains JCM 19037, JCM 19038, JCM 19039, and JCM 19055, Isolated from Aquatic Samples

**DOI:** 10.1128/genomeA.00622-14

**Published:** 2014-06-19

**Authors:** Toshiaki Kudo, Tomomi Nakahara, Xiaochi Zhang, Shigeto Taniyama, Osamu Arakawa, Shinji Murase, Hideaki Nakata, Kenshiro Oshima, Wataru Suda, Keiko Kitamura, Toshiya Iida, Yumi Oshida, Tetsushi Inoue, Yuichi Hongoh, Masahira Hattori, Moriya Ohkuma

**Affiliations:** a Graduate School of Fisheries Science and Environmental Studies, Nagasaki University, Nagasaki, Japan; b Graduate School of Science and Technology, Nagasaki University, Nagasaki, Japan; c Nagasaki Prefectural Institute of Fisheries, Nagasaki, Japan; d Center for Omics and Bioinformatics, Graduate School of Frontier Sciences, the University of Tokyo, Kashiwa, Japan; e Japan Collection of Microorganisms/Microbe Division, RIKEN BioResource Center, Tsukuba, Japan; f Department of Biological Sciences, Graduate School of Bioscience and Biotechnology, Tokyo Institute of Technology, Tokyo, Japan

## Abstract

Haloalkaliphilic strains JCM 19037, JCM 19038, JCM 19039, and JCM 19055, closely related to *Geomicrobium sediminis*, were isolated from aquatic samples, and their draft genome sequences were determined. The genome information of these four strains will be useful for studies of their physiology and ecology.

## GENOME ANNOUNCEMENT

The recently proposed genus *Geomicrobium* comprises Gram-positive, moderately haloalkaliphilic, non-spore-forming, motile or nonmotile bacteria (1, 2). Only two species in this genus have been described to date: *Geomicrobium halophilum*, isolated from soil (1), and *Geomicrobium sediminis*, isolated from marine sediment (2). During the course of our studies on bacterial diversity of various marine samples, we isolated four strains belonging to the genus *Geomicrobium* using the haloalkaliphilic culturing condition described previously (3). Strains F309, F386, and F414 were isolated from the intestine samples of the Japanese fire belly newt *Cynops ensicauda popei*, a tetrodotoxin-bearing animal, collected from a marsh in Okinawa, Japan. Tetrodotoxin is considered to accumulate in animals through the food web, starting with marine bacteria as primary sources (4). Strain U326 was isolated from a marine sediment sample collected from Omura Bay, Nagasaki, Japan (3). These four strains, F309, F386, F414, and U326, have been deposited at the Japan Collection of Microorganisms (JCM) (http://www.jcm.riken.jp) and are available as JCM 19037, JCM 19038, JCM 19039, and JCM 19055, respectively.

The genomes of *Geomicrobium* sp. strains JCM 19037, JCM 19038, JCM 19039, and JCM 19055 were sequenced using an Ion Torrent PGM System. The sequence reads of 387,337 for JCM 19037, 465,043 for JCM 19038, 435,174 for JCM 19039, and 431,208 for JCM 19055 were assembled using Newbler version 2.8 (Roche) into 75, 37, 57, and 48 contigs with *N*_50_ lengths of 86,630, 210,912, 137,361, and 144,705 bp, respectively. These assemblies resulted in draft genome sequences of 4,093,558 bp for JCM 19037, 3,872,620 bp for JCM 19038, 4,170,367 bp for JCM 19039, and 4,096,854 bp for JCM 19055, with 19×, 24×, 21×, and 21× redundancies and G+C contents of 44.3, 41.8, 44.2, and 41.5%, respectively. A total of 4,862, 4,251, 4,671, and 4,973 protein-coding genes and 58, 52, 59, and 60 RNA-encoding sequences for JCM 19037, JCM 19038, JCM 19039, and JCM 19055, respectively, were identified using the RAST server (5).

Phylogenetic analyses based on 16S rRNA gene sequence comparisons revealed that strains JCM 19037, JCM 19038, JCM 19039, and JCM 19055 were most closely related to the type strain of *Geomicrobium sediminis* (sequence identities, 98%, 99%, 97%, and 99%, respectively). Preliminary analyses of the genome sequences revealed the presence of genes relating to multi-subunit cation antiporter of alkaliphiles and ectoine biosynthesis of halophiles in each strain. Surprisingly, these four strains possessed a number of genes relating to dormancy and sporulation, although the genus *Geomicrobium* comprises non-spore-forming bacteria (1, 2). The genome information of these four strains will be useful for further studies of their physiology, taxonomy, and ecology.

### Nucleotide sequence accession numbers.

The genome sequences of *Geomicrobium* sp. strains JCM 19037, JCM 19038, JCM 19039, and JCM 19055 have been deposited at DDBJ/EMBL/GenBank database under the accession no. BAWZ01000001 to BAWZ01000075, BAXA01000001 to BAXA01000037, BAXB01000001 to BAXB01000057, and BAXL01000001 to BAXL01000048, respectively.
